# Effect of antenatal detection of small-for-gestational-age newborns in a risk stratified retrospective cohort

**DOI:** 10.1371/journal.pone.0224553

**Published:** 2019-10-31

**Authors:** Anna Kajdy, Jan Modzelewski, Monika Jakubiak, Artur Pokropek, Michał Rabijewski

**Affiliations:** 1 Centre of Postgraduate Medical Education, Department of Reproductive Health, Warsaw, Poland; 2 Institute of Philosophy and Sociology Polish Academy of Sciences, Warsaw, Poland; Stony Brook University Health Sciences Center School of Medicine, UNITED STATES

## Abstract

**Objective:**

Small-for-gestational-age (SGA) are neonates born with birth weight below the 10^th^ centile for a given week of pregnancy. It is a risk factor of perinatal and neonatal morbidity and mortality. There is an ongoing debate whether prenatal detection of SGA neonates is good predictor of perinatal outcome especially in low risk populations. Our primary aim was to compare the odds ratios for unfavorable outcome in a risk stratified cohort of SGA neonates in regard to prenatal detection status.

**Methods:**

This is a retrospective cohort study analysing the effect of prenatal detection on perinatal outcome. This cohort has been divided into a predefined low-risk and high-risk population. Electronic records of 39,032 singleton deliveries from 2010 through 2016 were analysed. SGA was defined as newborn weight below the 10^th^ percentile on the Fenton growth chart. Detected SGA (dSGA) neonates were those that were admitted for delivery with a prenatal ultrasound diagnosis of abnormal growth. Undetected SGA (uSGA) were neonates that were found to be below the 10^th^ percentile after birth. Perinatal and neonatal outcome was compared.

**Results:**

The detection rate in high-risk pregnancies was almost 45.7% versus low risk where it amounted to 18.9%. In both the high-risk and low-risk populations there was a significantly higher risk of composite mortality for undetected SGA compared to approporiate-for-gestational-age (AGA) (OR 7.95 CI 4.76–13.29; OR 14.4 CI 4.99–41.45 respectively). The odds for the composite neonatal outcome were significantly higher for dSGA and uSGA than for AGA in all the studied populations except for the uSGA in high risk population (OR 1.57 CI 0.97–3.53). Importantly, there was not a single case of intrauterine fetal death among detected SGA, in the low risk group.

**Conclusions:**

Prenatal detection of SGA status is related to perinatal outcomes, especially mortality. Therefore, assessment of SGA status even in low-risk pregnancies could help predict potential perinatal and neonatal complications.

## Introduction

Small-for-gestational-age (SGA) is a neonate with birth weight below the 10^th^ percentile. Antenatally all fetuses with an estimated weight below the 10^th^ percentile require monitoring. If the estimated weight falls below the 3^rd^ percentile or the fetus presents with abnormal Doppler results it is classified as growth restricted (FGR) [1]. Fetuses with normal Doppler indices and weight between 3–10 percentile are also classified as SGA prenatally [1]. All fetuses below the 10^th^ percentile regardless if growth restricted or not are at increased risk of neonatal morbidity (respiratory distress syndrome, intraventricular hemorrhage, seizures, sepsis) and mortality including risk of intrauterine fetal death (IUFD) and death within 28 days of birth [2]. Antenatal detection of SGA may play a role in preventing these sequalae. There is strong evidence for this relationship in high-risk pregnancies and serial screening is recommended in this group [3–6]. Although opinions differ, there are studies showing that detection in low risk pregnancies may also improve perinatal outcome [7–9]. Detection rates in the latter remain low and often fall below 15% [10–12]. Although >60% of unaffected SGA deliveries takes place at term, most guidelines and obstetric societies do not recommend ultrasound in late third trimester of pregnancy in low risk populations [2,4–6,13]. The primary aim of our study was to compare outcome of SGA neonates according to detection status in a risk stratified cohort. The study hypothesis was that detection improves outcome in both low and high-risk pregnancies. The secondary aim was to inspect detections rate in low and high-risk pregnancies.

## Materials and methods

Electronic database records of 39032 singleton deliveries that took place between the years 2010–2016 at the Saint Sophia Hospital, a tertiary unit in Warsaw, Poland were analysed retrospectively. Fenton Growth Chart was used to assess newborn weight. It is recommended for preterm born neonates, was assessed in previous studies, and is routinely used by neonatologist in Poland [14,15]. Live and stillborn neonates were grouped according to centiles. Birth weights for gestational age between 10-90^th^ percentiles were classified as appropriate-for gestational-age (AGA), below the 10^th^ SGA and those above the 90^th^ as large for gestational age (LGA). LGA neonates were excluded from the study. Other exclusion criteria were: neonates with abnormal karyotype, major congenital defects and infections. Minor congenital defects as defined by Eurocat were included in the study [16]. AGA group served as a control group for all the performed analysis. Detection status was based on maternal medical records. Neonates born to mother’s that had a prenatal diagnosis of SGA upon admission to the hospital were labelled detected (dSGA). Neonates born to mothers that did not have a prenatal ultrasound diagnosis upon admission to the hospital were labelled undetected (uSGA). Analysis of mode and time of detection was not the aim of this analysis. The primary interest was if the mere fact of being detected for smallness had any effect on outcome. We compared obstetric results of dSGA to uSGA both in all of the population, low-risk and high-risk populations. Low-risk included women age 18–40, 37–41 weeks gestational age, primiparas and multiparas (no more than 4 pregnancies), without diabetes mellitus (DM), gestational diabetes mellitus type 1(GDMG1), gestational diabetes mellitus type 2 (GDMG2) pregnancy hypertension (PH), pre-pregnancy hypertension (PPH), preeclampsia (PE), HELLP syndrome, pregnancy cholestasis, premature delivery, obesity, maternal smoking. Presence of at least one of the mentioned above criteria classified the pregnancy as high risk [17,18].

**Fig 1:**
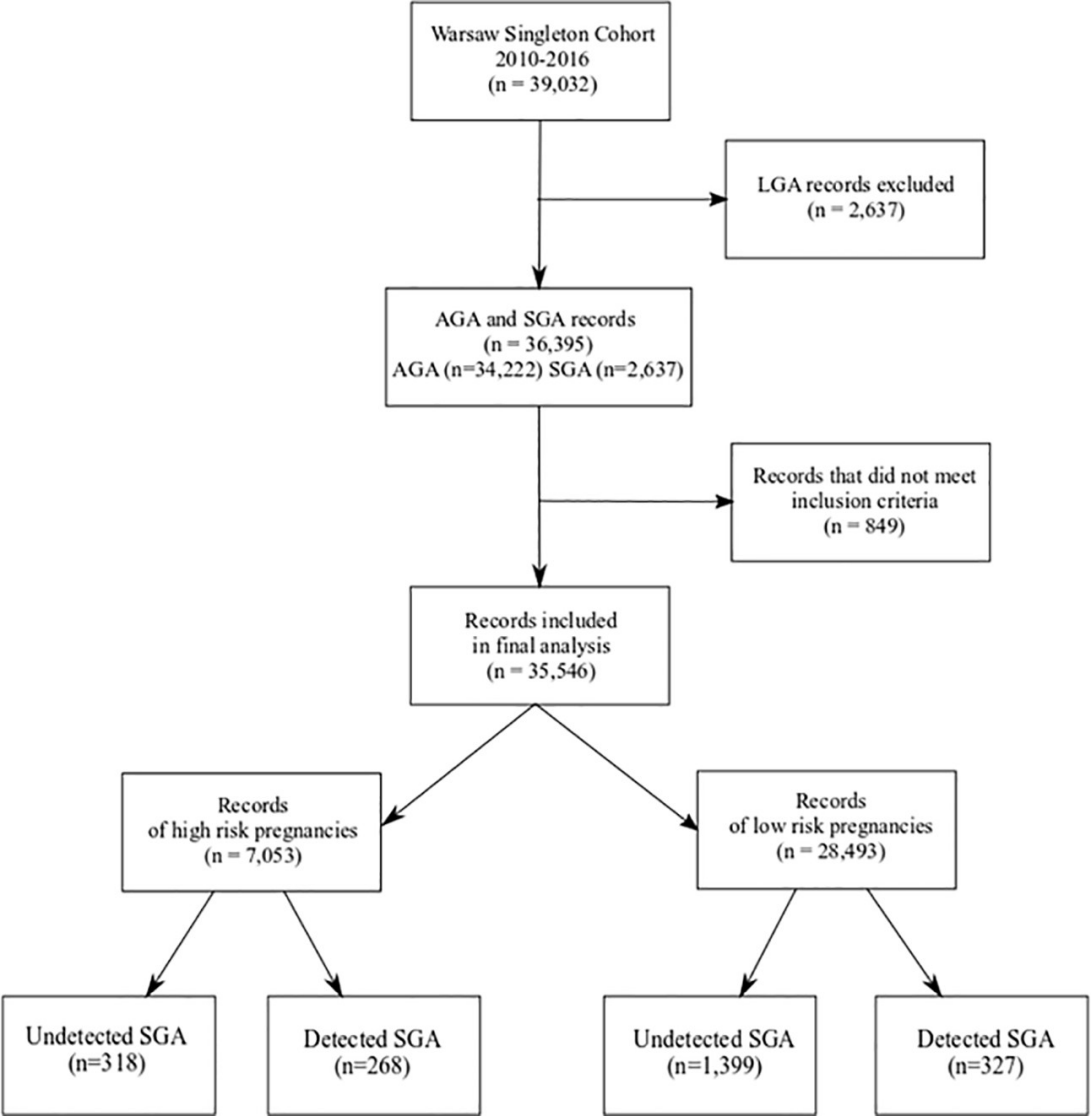
Flow diagram demonstrating the database extraction process. AGA, appropriate for gestational age; LGA–large for gestational age; SGA small for gestational age.

The cohort comprised of 2,312 SGA, 34,222 AGA and 2637 LGA neonates (5.6%, 87.6% and 6.7% respectively). LGA and 849 other records were excluded from the study according to the pre-established criteria described above. 35,546 records underwent final analysis (7,053 [19.8%] high-risk pregnancies and 28,493 [80.2%] low-risk pregnancies). 595 SGA neonates were diagnosed prenatally (overall detection rate 25.8%; 45.7% for high-risk pregnancies; 18.9% for low-risk pregnancies) (Fig 1).

All pregnant women upon admission had gestational age verified by last menstrual period (LMP), first trimester ultrasound or both [19].

Rates of the following neonatal complications were compared: neonatal death, necrotizing enterocolitis (NEC), sepsis, respiratory distress syndrome (RDS), Apgar < 7 in the 5^th^ minute, intraventricular haemorrhage (grade III and IV), documented seizures, leukomalacia and hypoglycaemia. These are rare complications therefore were combined into a composite neonatal outcome. The most severe outcomes analysed were IUFD and neonatal death, which were combined into composite mortality. Results were analysed for high-risk pregnancies, low-risk pregnancies and all of the population adjusted for pregnancy complications, maternal characteristics: maternal age, parity, HELLP, PH, PPH, PE, GDMG1, GDMG2, pregnancy cholestasis and preterm birth.

All calculations were performed using Stata 14.2 (StataCorp, College Station, TX). Two sets of results are presented: Crude odds ratio (OR) that reflect simple bivariate relations between outcome variables and SGA and adjusted OR (aOR) from multivariate logistic regression to control for confounding factors. For continuous outcomes linear regression was used. AGA pregnancies served as the referent group for all analyses. The reported results were obtained by maximum likelihood (ML) estimation with robust standard errors. In the article, we are reporting results obtained by maximum likelihood (ML) estimation. However, as outcome variables with rare events might cause a small-sample bias in the (ML) estimation for the robustness check, we used also penalized likelihood estimation. For our data, the later one shows no significant differences compared to ML [20]. Confidence intervals were produced at 95% level. Missing data: educational (2.9%), professional (2.1%) status, as well blood type (13%) and outcome variables: Apgar score 7 at 5 min; Respiratory distress syndrome; IVH grade III or IV; Neonatal sepsis; Periventricular leukomalacia; Confirmed seizures (16 cases for each outcome) were handled using the multiple imputation by chained equation (ICE) procedure [21]. The imputation model included all the analysed variables generating 10 imputed data sets. All estimates in multivariate models were obtained using multiple imputation methodology. This involved fitting five sets of models, each with one plausible value, and then combining these values using the Rubin rule [22].

Bioethics Committee of the Centre of Postgraduate Medical Education (reference number 47/PB/2018) approval for this project was obtained on 11.04.2018.

## Results

It was a primarily Caucasian population, with a defined marital status, higher education and high skill professions (Managers, Professionals, Technicians and Associate Professionals defined by ISCO08 codes) [23]. Tables 1, 2 and 3 present demographic details of the studied cohort for AGA and both SGA groups. In overview the risk of SGA was lower when the following characteristics were present: parity more than 2, older maternal age, being married, having higher education and having a high skill profession. There was a higher risk of SGA in relationship to smoking. Multivariate analysis (Table 1) confirms those results except for maternal age, marital status and high skill professions that were insignificant.

**Table 1:** Multivariate analysis of demographic characteristics. Logistic regression oefficients.

	dSGA			uSGA		
	aOR	CI	p	aOR	CI	p
Parity						
**1 (reference)**						
**2**	0.48	0.39–0.58	**0.000**	0.48	0.43–0.54	**0.000**
**3**	0.44	0.30–0.64	**0.000**	0.28	0.21–0.37	**0.000**
**4+**	0.24	0.11–0.56	**0.001**	0.54	0.37–0.78	**0.001**
Maternal Age (mean +/-SD)	1.13	1.03–1.23	**0.007**	1.02	0.97–1.08	0.484
Married (1 = yes; 0 = no)	0.92	0.75–1.12	0.387	0.92	0.81–1.03	0.149
Higher Education (1 = yes; 0 = no)	0.63	0.50–0.79	**0.000**	0.84	0.72–0.97	**0.018**
Professional (1 = yes; 0 = no)	0.97	0.82–1.15	0.728	0.97	0.88–1.08	0.628
Obesity	1.32	0.65–2.70	0.440	0.92	0.54–1.56	0.759
Maternal smoking	2.42	1.33–4.38	**0.004**	1.70	1.10–2.64	**0.017**

N = 35546; Number of imputations = 10; dSGA, detected small for gestational age; uSGA, undetected small for gestational age; SD, standard deviation. All statistically significant results are bolded.

**Table 2:** Summary of significant adjusted odds ratios of neonatal outcome for full population adjusted for socioeconomic variables, pregnancy complications, mode of labour.

	n AGA	n dSGA	aOR	CI	p	n uSGA	aOR	CI	p
All									
Apgar score 7 at 5 min	84	5.00	1.57	0.59–4.19	0.37	14.00	2.84	1.58–5.08	0.00
Respiratory distress syndrome	223	33.00	3.68	2.40–5.65	0.00	11.00	0.91	0.49–1.69	0.76
NICU admission	1,423	83.00	1.77	1.36–2.29	0.00	70.00	0.98	0.76–1.26	0.86
Time of NICU stay	6.58	20.93	2.06	1.84–2.29	0.00	10.37	0.47	0.35–0.58	0.00
Composite neonatal outcome	393	64.00	5.71	4.19–7.78	0.00	54.00	2.55	1.90–3.43	0.00
Neonatal death	24	3.00	4.54	1.20–17.14	0.03	6.00	5.81	2.26–14.92	0.00

**Table 3:** Univariate analysis of demographic characteristics.

	AGA	Detected SGA				Undetected SGA			
	N = 33.234	N = 595				N = 1.717			
	Value	Value	OR	CI	p	Value	OR	CI	p
Parity									
**1 (reference)**	17,806	421				1238			
**2**	11,993	138	0.50	0.42–0.61	**0.000**	396	0.48	0.43–0.54	**0.000**
**3**	2,609	30	0.51	0.35–0.74	**0.000**	51	0.28	0.21–0.38	**0.000**
**4+**	826	6	0.32	0.14–0.71	**0.005**	32	0.57	0.40–0.81	**0.002**
Maternal Age mean (SD)	31.48(4.23)	31.15 (4.72)	0.93	0.86–1.01	0.092	30.87(4.34)	0.87	0.82–0.91	**0.000**
Married (1 = yes; 0 = no)	26.941	452	0.75	0.62–0.90	**0.002**	1.320	0.78	0.70–0.88	**0.000**
Higher Education (1 = yes; 0 = no)	27,581	459	0.67	0.54–0.82	**0.000**	1384	0.84	0.74–0.96	**0.008**
Professional (1 = yes; 0 = no)	14,953	246	0.87	0.74–1.03	0.103	734	0.91	0.82–1.00	**0.048**
Obesity	281	8	1.60	0.79–3.24	0.195	15	1.02	0.59–0.61	1.723
Maternal smoking	229	12	2.83	1.58–5.09	**0.000**	23	1.89	1.23–2.91	**0.004**

This is a primarily Caucasian population. All statistically significant results are bolded. AGA, appropriate for gestational age; dSGA, detected small for gestational age; uSGA, undetected small for gestational age; SD, standard deviation

Risk of preterm birth was significantly higher for dSGA than for uSGA when calculated for all of the population. For uSGA the chances of being born preterm were also lower than for AGA, this result nearly reached statistical significance. This trend remained true when calculated separately for the high-risk population. (S1 Table, S2 Table)

Odds of induction versus spontaneous delivery were calculated for both groups and risk populations. In the low risk population, the OR for induction was higher both for dSGA and uSGA than for AGA, while OR for spontaneous delivery for both uSGA and dSGA were significantly lower than for AGA. This trend was not observed in the high-risk group.

In the high-risk group the OR for induction was 4 times lower than in the low risk group for dSGA in comparison to AGA. There was a statistically significant 3-fold higher risk of cesarean section for dSGA compared to AGA in the high-risk group. That tendency was observed regardless of risk but was twice as high for high-risk pregnancies. On the other hand, the chances for operative vaginal delivery among uSGA were two times higher than in the AGA group. (S2 Table, S3 Table)

The risk of cesarean section because of non-reassuring fetal heart rate patterns was very high and statistically significant regardless of pregnancy risk. Significant results were yielded for both dSGA and uSGA. In the high-risk population the OR was 6.64 (CI 5.12–8.61) and 2.58 (CI 1.87–3.34) and in the low risk population OR 4.35 (CI 3.35–5.64) and 2.19 (CI 1.86–2.57) respectively for dSGA and uSGA compared to AGA. (S1, S2 and S3 Tables)

In the high-risk population placental abruption was a significant complication of dSGA but not for uSGA. The OR for placental abruption was 6.03 (CI 3.31–11.0). That finding became insignificant in the low-risk population. (S2 Table)

GDMG1, PH, PPH, PE, HELLP had all OR greater than 1 for dSGA when calculated for the whole sample. In the high-risk population, the trend continued, except for PPH, which was not statistically significant. In the uSGA group OR for GDMG2, PH, PE and Cholestasis were significantly greater than for AGA. (S2 and S3 Tables)

**Fig 2:**
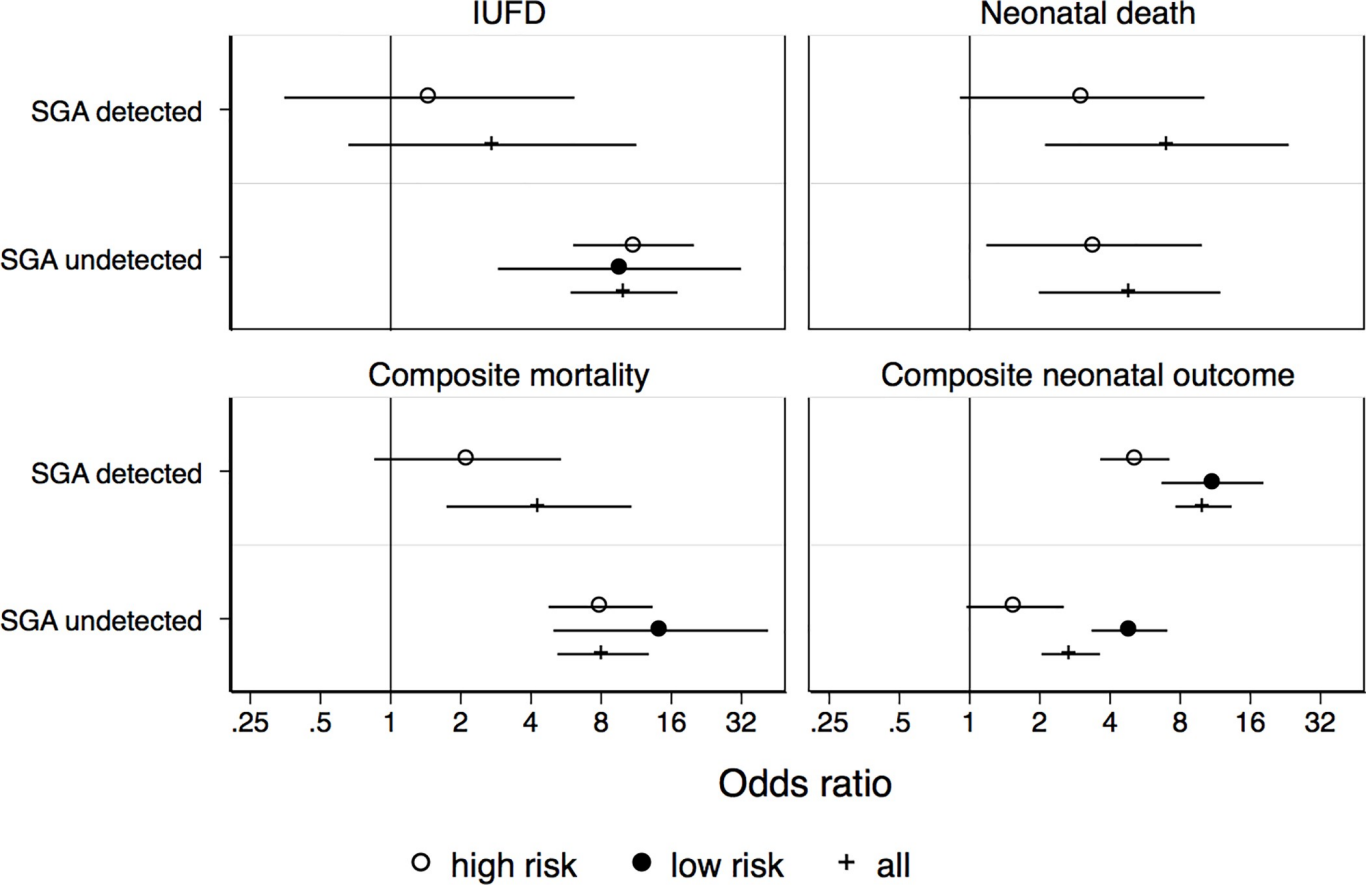
Odds ratios of mortality outcome and composite neonatal outcome presented in a risk stratified format. AGA; appropriate for gestational age newborns served as referent group for all calculations; IUFD, intrauterine fetal death; SGA, small for gestational age; Composite mortality: IUFD + neonatal death; Composite neonatal outcome: at least one incidence of neonatal death, necrotizing enterocolitis, sepsis, respiratory distress syndrome Apgar < 7 in the 5^th^ minute, intraventricular haemorrhage (grade III and IV), documented seizures or leukomalacia; All–the whole studied population; low risk population: women age 18–40, 37–41 weeks gestational age, primiparas and multiparas (no more than 4 pregnancies), without diabetes mellitus, gestational diabetes mellitus, pregnancy hypertension, pre-pregnancy hypertension, preeclampsia, HELLP syndrome, pregnancy cholestasis, premature delivery, obesity, maternal smoking.

IUFD was the main outcome of the study. The OR in the uSGA group was 10.02 (CI 5.91–17.00) versus 2.73 (CI 0.66–11.31) in the dSGA compared to AGA. In the high-risk population OR of IUFD in dSGA was higher than for AGA, but the result was not statistically significant (1.47 CI 0.35–6.14), while for uSGA odds of IUFD were 11 times greater than for AGA and statistically significant (11.01; CI 6.06–19.99). Similarly, for the low risk population odds of IUFD for uSGA were almost 10 times higher than for AGA (9.59; CI 2.88–31.89). In the low risk population there was not a single case of IUFD among dSGA (Fig 2).

To assess if detection changes the risk of death during the whole perinatal period and to increase the power of the analysis, the IUFD and neonatal death were combined into a single outcome called perinatal mortality. This yielded significant results for both the low risk and high-risk population as a group. In the high risk group, there was a significantly higher risk of composite mortality for uSGA than for AGA (OR 7.95 CI 4.76–13.29), but not for dSGA. That risk was even higher in the low risk population for uSGA (OR 14.4 CI 4.99–41.45) (Fig 2).

The odds of composite neonatal outcome were significantly higher for dSGA and uSGA than for AGA in all the studied populations (all, low-risk and high-risk) with uSGA in high risk population on the edge of statistical significance (OR 1.57 CI 0.97–3.53) (Fig 2). The ratio for NICU admissions was higher for the dSGA (all, high risk, low risk) but not for uSGA. The stay in the NICU was longer for the high risk dSGA and high risk uSGA. Respiratory distress syndrome was higher in dSGA but not in uSGA. Apgar score <7 was more frequent in the high risk dSGA and the uSGA. Apgar score <7 was more frequent in the high risk dSGA and the uSGA. Neonatal death was significantly more frequent in all dSGA and all and high risk uSGA (Fig 2 and S4 Table). The only other ORs that were found significant for neonatal outcome were NICU admission, length of stay in NICU and composite neonatal outcome for the dSGA and risk of neonatal death and perinatal mortality for uSGA. There was not a single neonatal death in the low-risk group (detected and undetected). (Fig 2)

## Discussion

Abnormal growth is a major risk factor of stillbirth [24]. Ego et al. used the RECODE classification to assess the role of abnormal growth as a risk factor of stillbirth. They concluded on the importance of improving prenatal detection in stillbirth prevention [25]. This observation was confirmed in works of Gardosi et al. [26,27]. Detection rates, especially among low risk populations are low [10]. Different strategies have been implemented to ameliorate detection, unfortunately thus far not yielding satisfactory results [13,28]. We present data from a country where such standard has not yet been developed. The overall detection rate was higher than that quoted by other authors [3,10,11]. Although it would be expected that with a national guideline implementing a growth scan in the general population at 28–32 weeks the detection rates should be even higher [29]. As showed in a prospective trial by Sovio et al. that is not necessarily the optimal solution [13]. Most guidelines do not recommend growth scans in low risk populations, albeit our population according to local guidelines did undergo at least one growth scan yielding a detection rate higher than that quoted by other authors, especially in the low risk population [2,4,6]. On the other hand, the prevalence of SGA is lower than that quoted in studies [1,7,9]. It should oscillate around 8%-10%, but it was only 5.6%. It may be the effect of the growth chart used. Perhaps the Fenton growth chart is not the optimal tool for assessing newborn weight in our population. Other studies showed that population-based growth charts are more effective at identifying both fetuses and newborns at risk of unfavourable outcome [30]. Even better results could be obtained by customisation of growth charts. This requires the development of coefficients for a specific population. Optimisation of prenatal and postnatal diagnosis is the key in ameliorating obstetric results [31,32]. Neither a prenatal nor neonatal population growth chart is established and implemented specifically for the Polish population. The presented cohort is very homogenous. There were no statistical differences in obesity, marital status, education, blood type or maternal smoking. In a univariate analysis the risk of smoking was slightly higher for both detected and undetected SGA compared to AGA, but a subsequent multivariate analysis did not confirm this significance. This might indicate that the smoking is only an indicator of sets of other health related risk behaviours captured by the effect of education. Educated people are usually more aware of potentially dangerous health behaviours like smoking, drinking alcohol and prefer better balanced diets [33].

The aim of the study was to assess if the fact of being identified prenatally as having abnormal growth regardless of timing and mode of detection had an impact on perinatal outcomes. In high risk pregnancies it is more likely to have out of recommendations extra scans to assess fetal wellbeing and growth. This is not recommended in Poland for low risk pregnancies. Hence, we looked if this impact was observed in two risk groups–low and high-risk.

The most essential outcome studied in the aspect of detecting SGA fetuses is risk of intrauterine death. Many studies did not manage to yield significant results when analysing this endpoint. The primary cause is the size of the studied cohorts. We managed to show that by detecting SGA fetuses we decrease the risk of intrauterine and neonatal death. That is a very significant finding because it provides evidence that screening in the general population, and not limiting it only to the high-risk population could have an important impact on perinatal mortality.

The odds ratio for composite neonatal outcome is higher in dSGA group than in uSGA (statistically significant in the high-risk population and for all of population adjusted for risk factors). This can be a result of two trends. First of all, detection is more likely in the high-risk population because of extra prenatal surveillance. The neonatal outcome in that group is a result of not only growth but also prematurity and other pregnancy complications. On the other hand, we can infer that detected were the more severe cases. Detected SGA were more likely not only to be born prematurely, but had a higher risk of caesarean section, interventions because of non-reassuring FHR and higher risk of placental abruption. Therefore, by detecting SGA fetuses we decreased the risk of perinatal mortality, not necessarily improving composite neonatal outcome. That may be the price of survival. On the other hand, in the low risk population detection resulted in not a single incidence of IUFD (Fig 2).

This was a retrospective cohort study, which obviously carries a risk of bias, and the authors are aware of that. Prospective trials, on the other hand, require very large groups to show statistical significance for mortality, especially in low risk populations. Such trials are very scarce and are very difficult to conduct. Our study was limited to one centre and involved a homogenous population in a large metropolitan area. It is the first study analysing smallness as a risk factor of outcome in a Polish population. Authors have not found a cohort of this size described for any other European population. Two American groups and one Australian have published two similar cohorts, and one significantly larger [8,9,30]. This study combines many aspects discussed by those authors. Two aspects represent the originality of our paper. First of all, it is the combination of the low and high-risk population within one maternity center; second of all we calculated not only the composite neonatal outcome but also perinatal mortality. Previous studies did not include stillborn neonates and therefore could not assess mortality risks [7,9]. Another potential limitation of our study is lack of ultrasound data regarding the timing of the last prenatal ultrasound. Due to the high educational and professional status of our cohort and local assessments of provision of medical care in our population, we can assume that the majority had prenatal care within the national guidelines which provides a growth scan at 28–32 weeks [29]. It could be argued that missing data regarding timing of ultrasound, estimated fetal weight or Doppler findings restricts the impact of our research. We stress that the aim of this paper was to assess whether prenatal diagnosis had an impact on outcome and how this effect is distributed among low-risk and high-risk populations. Despite those limitations our data confirms previously published findings but also presents new, especially in the aspect of perinatal mortality in the low risk population.

## Conclusions

Prenatal assessment of SGA status impacts perinatal outcomes, especially mortality regardless of predefined pregnancy risk. Our study shows that detection even in low-risk pregnancies predicts potential perinatal and neonatal complications. The optimal timing of the last ultrasound scan needs further prospective studies.

## Supporting information

S1 Table Perinatal outcomes odds ratios for all population. Calculations performed for all of the studied population. OR, odds ratio; CI, 95% Confidence interval; All statistically significant results are bolded. AGA, appropriate for gestational age; dSGA, detected small for gestational age; uSGA, undetected small for gestational age; IUFD, intrauterine fetal death; PGDM, diabetes mellitus; GDMG1, gestational diabetes mellitus treated with diet; GMG2 gestational diabetes mellitus treated with diet and insulin; PH, pregnancy hypertension, PPH, pre-pregnancy hypertension, PE preeclampsia, Preterm < 37 weeks gestation, obesity, BMI > 30; composite mortality: neonatal death + IUFD.
(DOCX)

S2 Table Perinatal outcomes odds ratios for high risk population. Calculations performed for high-risk population: after exclusion of low risk. OR, odds ratio; CI, 95% Confidence interval; All statistically significant results are bolded. AGA, appropriate for gestational age; dSGA, detected small for gestational age; uSGA, undetected small for gestational age; IUFD, intrauterine fetal death; PGDM, diabetes mellitus; GDMG1, gestational diabetes mellitus treated with diet; GMG2 gestational diabetes mellitus treated with diet and insulin; PH, pregnancy hypertension, PPH, pre-pregnancy hypertension, PE preeclampsia, Preterm < 37 weeks gestation, obesity, BMI > 30; composite mortality: neonatal death + IUFD.
(DOCX)

S3 Table Perinatal outcomes odds ratios for low risk population. Calculations performed for low-risk population: women age 18–40, 37–41 weeks gestational age, primiparas and multiparas (no more than 4 pregnancies), without diabetes mellitus, gestational diabetes mellitus, pregnancy hypertension, pre-pregnancy hypertension, preeclampsia, HELLP syndrome, pregnancy cholestasis, premature delivery, obesity, maternal smoking; OR, odds ratio; CI, 95% Confidence interval; All statistically significant results are bolded. AGA, appropriate for gestational age; dSGA, detected small for gestational age; uSGA, undetected small for gestational age; IUFD, intrauterine fetal death; composite mortality: neonatal death + IUFD.
(DOCX)

S4 Table Summary of significant odds ratios of neonatal outcome. Calculations performed for all of population, low risk and high risk according to obstetric criteria. Low-risk population: women age 18–40, 37–41 weeks gestational age, primiparas and multiparas (no more than 4 pregnancies), without diabetes mellitus, gestational diabetes mellitus, pregnancy hypertension, pre-pregnancy hypertension, preeclampsia, HELLP syndrome, pregnancy cholestasis, premature delivery, obesity, maternal smoking; OR, odds ratio; CI, 95% Confidence interval; All statistically significant results are bolded. AGA, appropriate for gestational age; dSGA, detected small for gestational age; uSGA, undetected small for gestational age; NICU–neonatal intensive care unit; composite neonatal outcome: at least one incidence of neonatal death, necrotizing enterocolitis, sepsis, respiratory distress syndrome Apgar < 7 in the 5^th^ minute, intraventricular haemorrhage (grade III and IV), documented seizures or leukomalacia; Neonatal death: death within 28 days of birth.
(DOCX)

## References

[1] Figueras F, Gratacós E. Update on the Diagnosis and Classification of Fetal Growth Restriction and Proposal of a Stage-Based Management Protocol. Fetal Diagn Ther. 2014;36(2):86–98. 10.1159/000357592 24457811

[2] American College of Obstetricians and Gynecologists. ACOG Practice bulletin no. 134: fetal growth restriction. Obstet Gynecol. 2013;121(5):1122–1133. 10.1097/01.AOG.0000429658.85846.f9 23635765

[3] Larsen T, Larsen JF, Petersen S, Greisen G. Detection of small-for-gestational-age fetuses by ultrasound screening in a high risk population: a randomized controlled study. *Br J Obstet Gynaecol*. 1992;99(6):469–474. 10.1111/j.1471-0528.1992.tb13783.x 1637761

[4] The Royal College of Obstetricians and Gynaecologists. The Investigation and Management of the Small–for–Gestational–Age Fetus. Green-Top Guidel. 2013;31 https://www.rcog.org.uk/globalassets/documents/guidelines/gtg_31.pdf. Accessed May 7, 2018.

[5] Institute of Obstetricians and Gynaecologists Royal of College of Phisicans of Ireland. Fetal growth restriction—recognition, diagnosis & managment. Clin Pract Guidel. 2017;28 https://www.hse.ie/eng/services/publications/clinical-strategy-and-programmes/fetal-growth-restriction.pdf. Accessed May 7, 2018.

[6] Lausman A, Kingdom J, Gagnon R, Basso M, Bos H, Crane J, et al Intrauterine Growth Restriction: Screening, Diagnosis, and Management. *J Obstet Gynaecol Can*. 2013;35(8):741–748. 10.1016/S1701-2163(15)30865-3 24007710

[7] Larkin J, Chauhan S, Simhan H. Small for Gestational Age: The Differential Mortality When Detected versus Undetected Antenatally. Am J Perinatol. 2016;34(04):409–414. 10.1055/s-0036-1592132 27627793

[8] Policiano C, Fonseca A, Mendes JM, Clode N, Graça LM. Small-for-gestational-age babies of low-risk term pregnancies: does antenatal detection matter? J Matern Fetal Neonatal Med. 4 2017:1–5. 10.1080/14767058.2017.1317741 28391748

[9] Mendez-Figueroa H, Truong VTT, Pedroza C, Khan AM, Chauhan SP. Small-for-gestational-age infants among uncomplicated pregnancies at term: a secondary analysis of 9 Maternal-Fetal Medicine Units Network studies. *Am J Obstet Gynecol*. 2016;215(5):628.e1–628.e7. 10.1016/j.ajog.2016.06.043 27372269

[10] Figueras F, Gardosi J. Intrauterine growth restriction: new concepts in antenatal surveillance, diagnosis, and management. *Am J Obstet Gynecol*. 2011;204(4):288–300. 10.1016/j.ajog.2010.08.055 21215383

[11] Hepburn M, Rosenberg K. An audit of the detection and management of small-for-gestational age babies. *Br J Obstet Gynaecol*. 1986;93(3):212–216. 10.1111/j.1471-0528.1986.tb07895.x 3964595

[12] Backe B, Nakling J. Effectiveness of antenatal care: a population based study. *Br J Obstet Gynaecol*. 1993;100(8):727–732. 10.1111/j.1471-0528.1993.tb14263.x 8399010

[13] Sovio U, White IR, Dacey A, Pasupathy D, Smith GCS. Screening for fetal growth restriction with universal third trimester ultrasonography in nulliparous women in the Pregnancy Outcome Prediction (POP) study: a prospective cohort study. *The Lancet*. 2015;386(10008):2089–2097. 10.1016/S0140-6736(15)00131-2 26360240 PMC4655320

[14] Fenton TR. A new growth chart for preterm babies: Babson and Benda’s chart updated with recent data and a new format. *BMC Pediatr*. 2003;3(1). 10.1186/1471-2431-3-13 14678563 PMC324406

[15] Fenton TR, Kim JH. A systematic review and meta-analysis to revise the Fenton growth chart for preterm infants. *BMC Pediatr*. 2013;13:59 10.1186/1471-2431-13-59 23601190 PMC3637477

[16] EUROCAT Central Registry. EUROCAT Guide 1.4: Instruction for the registration of congenital anomalies. http://www.eurocat-network.eu/content/Full%20Guide%201%204%20version%2020_Dec2017_clean.pdf. Accessed May 7, 2018.

[17] Pray L, ed. An Update on Research Issues in the Assessment of Birth Settings: Workshop Summary. Washington: National Academies Press; 2013. http://www.ncbi.nlm.nih.gov/books/NBK201937/. Accessed May 7, 2018. 24851306

[18] Figueras F, Gratacos E, Rial M, Gull I, Krofta L, Lubusky M, et al Revealed versus concealed criteria for placental insufficiency in an unselected obstetric population in late pregnancy (RATIO37): randomised controlled trial study protocol. *BMJ Open*. 2017;7(6). 10.1136/bmjopen-2016-014835 28619771 PMC5623458

[19] Butt K, Lim K, Lim K, Bly S, Butt K, Cargill Y, et al Determination of Gestational Age by Ultrasound. J Obstet Gynaecol Can. 2014;36(2):171–181. 10.1016/S1701-2163(15)30664-2 24518917

[20] Cole SR, Chu H, Greenland S. Maximum Likelihood, Profile Likelihood, and Penalized Likelihood: A Primer. *Am J Epidemiol*. 2014;179(2):252–260. 10.1093/aje/kwt245 24173548 PMC3873110

[21] Royston Patrick. Multiple imputation of missing values. *Stata J* 2004 4 *Number* 3 *Pp* 227–241.:15.

[22] Little R. and Rubin D. (1987) Statistical Analysis with Missing Data. John Wiley and Sons Publishers, New York.

[23] ILO (2012). International Standard Classification of Occupations. Geneva: International Labour Office Retrieved from http://www.ilo.org/wcmsp5/groups/public/—dgreports/—dcomm/—publ/documents/publication/wcms_172572.pdf.

[24] Winbo I, Serenius F, Dahlquist G, Källén B. Maternal risk factors for cause-specific stillbirth and neonatal death. *Acta Obstet Gynecol Scand*. 2001;80(3):235–244. 10.1034/j.1600-0412.2001.080003235.x 11207489

[25] Ego A, Zeitlin J, Batailler P, Cornec S, Fondeur A, Baran-Marszak M, et al Stillbirth classification in population-based data and role of fetal growth restriction: the example of RECODE. *BMC Pregnancy Childbirth*. 2013;13(1):182. 24090495 10.1186/1471-2393-13-182 PMC3850812

[26] Gardosi J, Madurasinghe V, Williams M, Malik A, Francis A. Maternal and fetal risk factors for stillbirth: population based study. *BMJ*. 2013;346(jan24 3):f108–f108. 10.1136/bmj.f108 23349424 PMC3554866

[27] Gardosi J, Giddings S, Clifford S, Wood L, Francis A. Association between reduced stillbirth rates in England and regional uptake of accreditation training in customised fetal growth assessment. *BMJ Open*. 2013;3(12):e003942 10.1136/bmjopen-2013-003942 24345900 PMC3884620

[28] Gaillard R, Jaddoe VWV. Assessment of Fetal Growth by Customized Growth Charts. Ann Nutr Metab. 2014;65(2–3):149–155. 10.1159/000361055 25413653

[29] Polish Society of Obstetrics and Gynecology. Recommendations of the Polish Society of Obstetrics and Gynecology for prenatal care in normal pregnancy. Ginekol Pol. 2005;76(7):517–527. 16363377

[30] Madden JV, Flatley CJ, Kumar S. Term small-for-gestational-age infants from low-risk women are at significantly greater risk of adverse neonatal outcomes. *Am J Obstet Gynecol*. 2018;218(5):525.e1–525.e9. 10.1016/j.ajog.2018.02.008 29462628

[31] Narchi H, Skinner A, Williams B. Small for gestational age neonates–are we missing some by only using standard population growth standards and does it matter? *J Matern Fetal Neonatal Med*. 2010;23(1):48–54. 10.3109/14767050903067352 19565425

[32] Agarwal P, Rajadurai VS, Yap F, Yeo G, Chong YS, Kwek K, et al Comparison of customized and cohort-based birthweight standards in identification of growth-restricted infants in GUSTO cohort study. *J Matern Fetal Neonatal Med*. October 2015:1–4. 10.3109/14767058.2015.1092956 26366791

[33] Borgonovi F. A life-cycle approach to the analysis of the relationship between social capital and health in Britain. *Soc Sci Med 1982*. 2010;71(11):1927–1934. 10.1016/j.socscimed.2010.09.018 20943301

