# Supplementary material for: Effect of antenatal detection of small-for-gestational-age newborns in a risk stratified retrospective cohort
Source: PLoS One. 2019 Oct 31;14(10):e0224553. doi: 10.1371/journal.pone.0224553 (PMC6822749; doi:10.1371/journal.pone.0224553)
Supplement: S1 Table — Calculations performed for all of the studied population. OR, odds ratio; CI, 95% Confidence interval; All statistically significant results are bolded. AGA, appropriate for gestational age; dSGA, detected small for gestational age; uSGA, undetected small for gestational age; IUFD, intrauterine fetal death; PGDM, diabetes mellitus; GDMG1, gestational diabetes mellitus treated with diet; GMG2 gestational diabetes mellitus treated with diet and insulin; PH, pregnancy hypertension, PPH, pre-pregnancy hypertension, PE preeclampsia, Preterm < 37 weeks gestation, obesity, BMI > 30; composite mortality: neonatal death + IUFD. (DOCX) [file pone.0224553.s001.docx]

**S1 Table. Perinatal outcomes odds ratios for all population**

|  | n AGA | n dSGA | OR | CI | p | n uSGA | OR | CI | p |
| --- | --- | --- | --- | --- | --- | --- | --- | --- | --- |
| Pregnancy complications | | | | | | | | | |
| GDMG1 | 1,947 | 49 | 1.44 | 1.07- 1.94 | **0.02** | 92 | 0.91 | 0.73- 1.13 | 0.39 |
| GDMG2 | 385 | 11 | 1.61 | 0.88- 2.94 | 0.12 | 10 | 0.50 | 0.27- 0.94 | **0.03** |
| PGDM | 14 | 1 | 3.99 | 0.52- 30.43 | 0.18 | 0 | 1.00 | n/a | n/a |
| PH | 1,,090 | 77 | 4.38 | 3.42- 5.61 | **0.00** | 72 | 1.29 | 1.01- 1.65 | **0.04** |
| PPH | 229 | 10 | 2.46 | 1.30- 4.67 | **0.01** | 6 | 0.51 | 0.22- 1.14 | 0.10 |
| PE | 87 | 26 | 17.41 | 11.15- 27.19 | **0.00** | 12 | 2.68 | 1.46- 4.91 | **0.00** |
| HELLP | 28 | 10 | 20.27 | 9.80- 41.93 | **0.00** | 3 | 2.08 | 0.63- 6.83 | 0.23 |
| Cholestasis | 406 | 7 | 0.96 | 0.45- 2.04 | 0.92 | 10 | 0.47 | 0.25- 0.89 | **0.02** |
| Preterm | 1,380 | 142 | 7.24 | 5.95- 8.80 | **0.00** | 56 | 0.78 | 0.59- 1.02 | 0.07 |
| Mode of Labor | | | | | | | | | |
| Induced or augmented labor | 7,503 | 169 | 1.36 | 1.14- 1.63 | **0.00** | 485 | 1.35 | 1.21- 1.50 | **0.00** |
| Spontaneous | 25,731 | 426 | 0.74 | 0.61- 0.88 | **0.00** | 1232 | 0.74 | 0.66- 0.83 | **0.00** |
| Route of delivery | | | | | | | | | |
| Vaginal | 23,225 | 234 | 0.28 | 0.24- 0.33 | **0.00** | 1224 | 1.07 | 0.96- 1.19 | 0.22 |
| Cesarean section | 9,457 | 350 | 3.59 | 3.05- 4.24 | **0.00** | 436 | 0.86 | 0.77- 0.96 | **0.01** |
| Operative vaginal delivery | 552 | 11 | 1.12 | 0.61- 2.04 | 0.72 | 57 | 2.03 | 1.54- 2.68 | **0.00** |
| Indication for cesarean section | | | | | | | | | |
| placental abruption | 113 | 12.00 | 6.03 | 3.31- 11.00 | **0.00** | 6 | 1.03 | 0.45- 2.34 | 0.95 |
| Failed trial of labor | 1,216 | 15 | 0.68 | 0.41- 1.14 | 0.14 | 53 | 0.84 | 0.63- 1.11 | 0.22 |
| Non reassuring FHR | 2,315 | 181 | 5.84 | 4.88- 6.99 | **0.00** | 247 | 2.24 | 1.95- 2.58 | **0.00** |
| Other | 3,997 | 92 | 1.33 | 1.06- 1.66 | **0.01** | 125 | 0.57 | 0.47- 0.69 | **0.00** |
| Perinatal mortality | | | | | | | | | |
| Composite mortality | 65 | 5 | 4.32 | 1.74- 10.78 | **0.00** | 27 | 8.15 | 5.19-12.80 | **0.00** |
| IUFD | 41 | 2 | 2.73 | 0.66- 11.31 | 0.17 | 21 | 10.02 | 5.91-17.00 | **0.00** |

Calculations performed for all of the studied population. OR, odds ratio; CI, 95% Confidence interval; All statistically significant results are bolded. AGA, appropriate for gestational age; dSGA, detected small for gestational age; uSGA, undetected small for gestational age; IUFD, intrauterine fetal death; PGDM, diabetes mellitus; GDMG1, gestational diabetes mellitus treated with diet; GMG2 gestational diabetes mellitus treated with diet and insulin; PH, pregnancy hypertension, PPH, pre-pregnancy hypertension, PE preeclampsia, Preterm < 37 weeks gestation, obesity, BMI > 30; composite mortality: neonatal death + IUFD
