# Supplementary material for: Effect of antenatal detection of small-for-gestational-age newborns in a risk stratified retrospective cohort
Source: PLoS One. 2019 Oct 31;14(10):e0224553. doi: 10.1371/journal.pone.0224553 (PMC6822749; doi:10.1371/journal.pone.0224553)
Supplement: S2 Table — Calculations performed for high-risk population: after exclusion of low risk. OR, odds ratio; CI, 95% Confidence interval; All statistically significant results are bolded. AGA, appropriate for gestational age; dSGA, detected small for gestational age; uSGA, undetected small for gestational age; IUFD, intrauterine fetal death; PGDM, diabetes mellitus; GDMG1, gestational diabetes mellitus treated with diet; GMG2 gestational diabetes mellitus treated with diet and insulin; PH, pregnancy hypertension, PPH, pre-pregnancy hypertension, PE preeclampsia, Preterm < 37 weeks gestation, obesity, BMI > 30; composite mortality: neonatal death + IUFD. (DOCX) [file pone.0224553.s002.docx]

**S2 Table. Perinatal outcomes odds ratios for high risk population**

|  | n AGA | n dSGA | OR | CI | p | n uSGA | OR | CI | p |
| --- | --- | --- | --- | --- | --- | --- | --- | --- | --- |
| Pregnancy complications | | | | | | | | | |
| GDMG1 | 1,947 | 49 | 0.52 | 0.38-0.71 | **0.00** | 92 | 0.95 | 0.74-1.21 | 0.66 |
| GDMG2 | 385 | 11 | 0.68 | 0.37-1.25 | 0.21 | 10 | 0.51 | 0.27-0.97 | **0.04** |
| PGDM | 14 | 1 | 1.73 | 0.23-13.18 | 0.60 | 0 | 1.00 | n/a | n/a |
| PH | 1,090 | 77 | 1.99 | 1.51-2.61 | **0.00** | 72 | 1.44 | 1.10-1.89 | **0.01** |
| PPH | 229 | 10 | 1.06 | 0.55-2.01 | 0.87 | 6 | 0.52 | 0.23-1.19 | 0.12 |
| PE | 87 | 26 | 7.88 | 4.99-12.44 | **0.00** | 12 | 2.88 | 1.56-5.32 | **0.00** |
| HELLP | 28 | 10 | 8.91 | 4.28-18.55 | **0.00** | 3 | 2.19 | 0.66-7.24 | 0.20 |
| Cholestasis | 406 | 7 | 0.40 | 0.19-0.85 | **0.02** | 10 | 0.48 | 0.26-0.92 | **0.03** |
| Preterm | 1,380 | 142 | 4.15 | 3.24-5.32 | **0.00** | 56 | 0.79 | 0.59-1.06 | 0.11 |
| Mode of Labor | | | | | | | | | |
| Induced or augmented labor | 1,786 | 46 | 0.54 | 0.39-0.75 | **0.00** | 103 | 1.26 | 0.99-1.60 | 0.06 |
| Spontaneous | 4,681 | 222 | 1.84 | 1.33-2.54 | **0.00** | 215 | 0.80 | 0.63-1.01 | 0.06 |
| Route of delivery | | | | | | | | | |
| Vaginal | 3,982 | 62 | 0.19 | 0.14-0.25 | **0.00** | 190 | 0.93 | 0.74-1.17 | 0.51 |
| Cesarean section | 2,412 | 203 | 5.25 | 3.95-6.97 | **0.00** | 120 | 1.02 | 0.81-1.29 | 0.87 |
| Operative vaginal delivery | 73 | 3 | 0.99 | 0.31-3.17 | 0.99 | 8 | 2.26 | 1.08-4.73 | **0.03** |
| Indication for cesarean section | | | | | | | | | |
| Placental abruption | 51 | 10 | 4.88 | 2.45-9.71 | **0.00** | 2 | 0.80 | 0.19-3.29 | 0.75 |
| Failed trial of labor | 233 | 7 | 0.72 | 0.33-1.54 | 0.39 | 12 | 1.05 | 0.58-1.90 | 0.87 |
| Non reassuring FHR | 572 | 105 | 6.64 | 5.12-8.61 | **0.00** | 62 | 2.50 | 1.87-3.34 | **0.00** |
| other | 800 | 41 | 1.27 | 0.90-1.79 | 0.17 | 26 | 0.63 | 0.42-0.94 | **0.02** |
| Perinatal mortality | | | | | | | | | |
| Composite mortality | 57 | 5 | 2.14 | 0.85-5.38 | 0.11 | 21 | 7.95 | 4.76-13.29 | **0.00** |
| IUFD | 33 | 2 | 1.47 | 0.35-6.14 | 0.60 | 17 | 11.01 | 6.06-19.99 | **0.00** |

Calculations performed for high-risk population: after exclusion of low risk. OR, odds ratio; CI, 95% Confidence interval; All statistically significant results are bolded. AGA, appropriate for gestational age; dSGA, detected small for gestational age; uSGA, undetected small for gestational age; IUFD, intrauterine fetal death; PGDM, diabetes mellitus; GDMG1, gestational diabetes mellitus treated with diet; GMG2 gestational diabetes mellitus treated with diet and insulin; PH, pregnancy hypertension, PPH, pre-pregnancy hypertension, PE preeclampsia, Preterm < 37 weeks gestation, obesity, BMI > 30; composite mortality: neonatal death + IUFD
