# Supplementary material for: Effect of antenatal detection of small-for-gestational-age newborns in a risk stratified retrospective cohort
Source: PLoS One. 2019 Oct 31;14(10):e0224553. doi: 10.1371/journal.pone.0224553 (PMC6822749; doi:10.1371/journal.pone.0224553)
Supplement: S3 Table — Calculations performed for low-risk population: women age 18–40, 37–41 weeks gestational age, primiparas and multiparas (no more than 4 pregnancies), without diabetes mellitus, gestational diabetes mellitus, pregnancy hypertension, pre-pregnancy hypertension, preeclampsia, HELLP syndrome, pregnancy cholestasis, premature delivery, obesity, maternal smoking; OR, odds ratio; CI, 95% Confidence interval; All statistically significant results are bolded. AGA, appropriate for gestational age; dSGA, detected small for gestational age; uSGA, undetected small for gestational age; IUFD, intrauterine fetal death; composite mortality: neonatal death + IUFD. (DOCX) [file pone.0224553.s003.docx]

**S3 Table. Perinatal outcomes odds ratios for low risk population**

|  | n AGA | n dSGA | OR | CI | p | n uSGA | OR | CI | p |
| --- | --- | --- | --- | --- | --- | --- | --- | --- | --- |
| Mode of Labor | | | | | | | | | |
| Induced or augmented labour | 5,717 | 123.00 | 2.22 | 1.77-2.78 | **0.00** | 382 | 1.38 | 1.23-1.56 | **0.00** |
| Spontaneous | 21,050 | 204.00 | 0.45 | 0.36-0.56 | **0.00** | 1017 | 0.72 | 0.64-0.82 | **0.00** |
| Route of delivery | | | | | | | | | |
| Vaginal | 19,243 | 172.00 | 0.43 | 0.35-0.54 | **0.00** | 1034 | 1.11 | 0.98-1.25 | 0.10 |
| Cesarean section | 7,045 | 147.00 | 2.29 | 1.84-2.85 | **0.00** | 316 | 0.82 | 0.72-0.93 | **0.00** |
| Operative vaginal delivery | 479 | 8.00 | 1.38 | 0.68-2.79 | 0.38 | 49 | 1.99 | 1.48-2.69 | **0.00** |
| Indication for cesarean section | | | | | | | | | |
| Placental abruption | 62 | 2 | 2.65 | 0.65-10.88 | 0.18 | 4 | 1.24 | 0.45-3.40 | 0.68 |
| Failed trial of labor | 983 | 8 | 0.66 | 0.32-1.33 | 0.24 | 41 | 0.79 | 0.58-1.09 | 0.15 |
| Non reassuring FHR | 1,743 | 76 | 4.35 | 3.35-5.64 | **0.00** | 185 | 2.19 | 1.86-2.57 | **0.00** |
| Other | 3,197 | 51 | 1.35 | 0.99-1.83 | **0.05** | 99 | 0.56 | 0.45-0.69 | **0.00** |
| Perinatal mortality | | | | | | | | | |
| Composite mortality | 8 | 0 | n/a | n/a | n/a | 6 | 14.41 | 4.99-41.58 | **0.00** |
| IUFD | 8 | 0 | n/a | n/a | n/a | 4 | 9.59 | 2.88-31.89 | **0.00** |

Calculations performed for low-risk population: women age 18-40, 37-41 weeks gestational age, primiparas and multiparas (no more than 4 pregnancies), without diabetes mellitus, gestational diabetes mellitus, pregnancy hypertension, pre-pregnancy hypertension, preeclampsia, HELLP syndrome, pregnancy cholestasis, premature delivery, obesity, maternal smoking; OR, odds ratio; CI, 95% Confidence interval; All statistically significant results are bolded. AGA, appropriate for gestational age; dSGA, detected small for gestational age; uSGA, undetected small for gestational age; IUFD, intrauterine fetal death; composite mortality: neonatal death + IUFD
