# Supplementary material for: Effect of antenatal detection of small-for-gestational-age newborns in a risk stratified retrospective cohort
Source: PLoS One. 2019 Oct 31;14(10):e0224553. doi: 10.1371/journal.pone.0224553 (PMC6822749; doi:10.1371/journal.pone.0224553)
Supplement: S4 Table — Calculations performed for all of population, low risk and high risk according to obstetric criteria. Low-risk population: women age 18–40, 37–41 weeks gestational age, primiparas and multiparas (no more than 4 pregnancies), without diabetes mellitus, gestational diabetes mellitus, pregnancy hypertension, pre-pregnancy hypertension, preeclampsia, HELLP syndrome, pregnancy cholestasis, premature delivery, obesity, maternal smoking; OR, odds ratio; CI, 95% Confidence interval; All statistically significant results are bolded. AGA, appropriate for gestational age; dSGA, detected small for gestational age; uSGA, undetected small for gestational age; NICU–neonatal intensive care unit; composite neonatal outcome: at least one incidence of neonatal death, necrotizing enterocolitis, sepsis, respiratory distress syndrome Apgar < 7 in the 5th minute, intraventricular haemorrhage (grade III and IV), documented seizures or leukomalacia; Neonatal death: death within 28 days of birth. (DOCX) [file pone.0224553.s004.docx]

**S4 Table. Summary of significant odds ratios of neonatal outcome**

|  | n AGA | n dSGA | OR | CI | p | n uSGA | OR | CI | p |
| --- | --- | --- | --- | --- | --- | --- | --- | --- | --- |
| All | | | | | | | | | |
| Apgar score 7 at 5 min | 84 | 5 | 3.34 | 1.35-8.27 | **0.01** | 14 | 3.25 | 1.84-5.74 | **0.00** |
| Respiratory distress syndrome | 223 | 33 | 8.69 | 5.97-12.64 | **0.00** | 11 | 0.96 | 0.52-1.76 | 0.89 |
| NICU admission | 1,423 | 83 | 3.62 | 2.86-4.60 | **0.00** | 70 | 0.95 | 0.74-1.21 | 0.68 |
| Time of NICU stay* | 6.58 | 20.93 | 14.35 | 12.07-16.64 | **0.00** | 10.37 | 3.79 | 1.32-6.27 | **0.00** |
| Composite neonatal outcome | 393 | 64 | 10.07 | 7.63-13.30 | **0.00** | 54 | 2.71 | 2.03-3.62 | **0.00** |
| Neonatal death | 24 | 3 | 7.01 | 2.11-23.35 | **0.00** | 6 | 4.85 | 1.98-11.89 | **0.00** |
| Low risk | | | | | | | | | |
| Apgar score 7 at 5 min | 39 | 0 | n/a | n/a | n/a | 7 | 3.45 | 1.54-7.72 | **0.00** |
| NICU admission | 914 | 18 | 1.65 | 1.019-2.66 | **0.04** | 42 | 0.88 | 0.64-1.20 | **0.43** |
| Time of NICU stay* | 4.14 | 5.23 | 1.10 | -0.24-2.43 | 0.11 | 4.64 | 0.50 | -0.38-1.39 | **0.04** |
| Composite neonatal outcome | 141 | 18 | 11.00 | 6.65-18.19 | **0.00** | 35 | 4.85 | 3.33-7.04 | **0.00** |
| Neonatal death | 0 | 0 | n/a | n/a | n/a | 2 | n/a | n/a | n/a |
| High risk | | | | | | | | | |
| Apgar score 7 at 5 min | 45 | 5 | 2.71 | 1.07-6.89 | **0.04** | 7 | 3.24 | 1.45-7.25 | **0.00** |
| Respiratory distress syndrome | 181 | 32 | 4.71 | 3.16-7.00 | **0.00** | 7 | 0.79 | 0.37-1.69 | 0.54 |
| NICU admission | 509 | 65 | 3.75 | 2.79-5.03 | **0.00** | 28 | 1.13 | 0.76-1.68 | 0.55 |
| Time of NICU stay* | 10.96 | 25.28 | 14.32 | 10.44-18.19 | **0.00** | 18.97 | 8.00 | 2.29-13.71 | **0.01** |
| Composite neonatal outcome | 252 | 46 | 5.11 | 3.63-7.19 | **0.00** | 19 | 1.57 | 0.97-2.53 | 0.07 |
| Neonatal death | 24 | 3 | 3.04 | 0.91-10.16 | 0.07 | 4 | 3.42 | 1.18-9.92 | **0.04** |

Calculations performed for all of population, low risk and high risk according to obstetric criteria. Low-risk population: women age 18-40, 37-41 weeks gestational age, primiparas and multiparas (no more than 4 pregnancies), without diabetes mellitus, gestational diabetes mellitus, pregnancy hypertension, pre-pregnancy hypertension, preeclampsia, HELLP syndrome, pregnancy cholestasis, premature delivery, obesity, maternal smoking; OR, odds ratio; CI, 95% Confidence interval; All statistically significant results are bolded. AGA, appropriate for gestational age; dSGA, detected small for gestational age; uSGA, undetected small for gestational age; NICU – neonatal intensive care unit; composite neonatal outcome: at least one incidence of neonatal death, necrotizing enterocolitis, sepsis, respiratory distress syndrome Apgar < 7 in the 5^th^ minute, intraventricular haemorrhage (grade III and IV), documented seizures or leukomalacia; Neonatal death: death within 28 days of birth.

* Continuous outcome: differences in natural metric instead of OR, means in groups instead of number of cases.
